# Development Level and Obstacle Factors of China’s Marine Food Production System

**DOI:** 10.3390/foods15061031

**Published:** 2026-03-16

**Authors:** Haotian Tong, Xiaoting Zhang, Enjun Xia, Cong Sun, Jieping Huang

**Affiliations:** School of Management, Beijing Institute of Technology, Beijing 100081, China; tonghaotian@bit.edu.cn (H.T.); zxting@bit.edu.cn (X.Z.); enjunxia@bit.edu.cn (E.X.); congsun@bit.edu.cn (C.S.)

**Keywords:** marine food production system, text analysis, fuzzy comprehensive evaluation, entropy weight method, obstacle degree model, spatiotemporal differentiation characteristics

## Abstract

The development of China’s marine food production system is receiving increasing attention, as its developmental level and obstacle factors will profoundly impact the nation’s future food security and nutritional supply. This study establishes a theoretical framework for evaluating the development level of marine food production systems based on three dimensions—resources, benefits, and governance—structured around the logical framework of “exogenous safeguard, endogenous drive, goal oriented”. First, a three-tier coding method based on grounded theory was employed to construct a Chinese marine food production system evaluation framework encompassing 28 specific indicators. Subsequently, a comprehensive weighting of these indicators was achieved by integrating fuzzy comprehensive evaluation with the entropy weighting method. Finally, based on the evaluation results and obstacle degree modeling, a comprehensive assessment study was conducted on 11 coastal provinces and cities, focusing on developmental level investigation and obstacle factor analysis. The results indicate that China’s marine food production system development level exhibits a trend of slow, fluctuating growth overall, maintaining an average annual growth rate of 3.23%. However, significant differentiation characteristics are emerging, with high regional heterogeneity and substantial variation in obstacle factors. Currently, the main constraints hindering the development of the marine food production system are insufficient human resource supply, uneven production resource distribution (higher in the north, lower in the south), and intensified fluctuations in comprehensive output. Finally, this study proposes three strategic recommendations: ecological restoration coupled with strict controls, comprehensive restructuring of the human resource support system, and establishing a multi-scale comprehensive evaluation mechanism. These strategies aim to disrupt the transmission mechanisms of different obstacle factors and accelerate the rapid development of the marine food production system.

## 1. Introduction

Feeding 22% of the world’s population with just 7% of its arable land has long been a source of pride for China [1], yet beneath this prosperity lie hidden concerns: First, terrestrial agriculture operates under sustained pressure, with factors like imbalanced land reclamation and excessive resource depletion pushing land-based food production near its limits [2]. Second, reliance on imports is growing. For instance, soybean imports accounted for 81.05% of China’s total in 2023, while beef imports reached 28.20%. Third, rising consumption of meat, seafood, and dairy products alongside declining grain consumption places greater strain on China’s food production system, as meat consumption requires more agricultural land to support feed supply [3,4]. China is developing its agricultural system based on a “comprehensive food perspective,” advocating for food acquisition through multiple channels. The marine food production system is a key focus area, playing a vital role in ensuring national nutrition and food security [5]. For China, considering the development of marine food production systems is of significant importance for refining the national food security strategy [6].

Given that China’s deep-sea aquaculture is currently in the demonstration and initial stages, the core of China’s marine food production system is actually composed of coastal and offshore fishing alongside coastal aquaculture [7], exhibiting four key characteristics. First, “coastal operations outweigh offshore ones.” This is reflected in the fact that China’s total coastal fishing and coastal aquaculture output far exceeds that of offshore fishing and aquaculture, with a production ratio of approximately 10:1 [8]. While this reduces production difficulties in terms of geographical distance, it increases conservation challenges at the resource and environmental level, as the environmental impact of industrial activities more readily affects coastal food production. Second, “aquaculture outweighs capture fisheries.” China currently boasts the world’s highest marine aquaculture output [9]. In the first quarter of 2024, China’s marine capture fisheries yielded 2.1689 million tons, while marine aquaculture produced 4.0225 million tons. Marine aquaculture offers greater controllability than fishing, significantly boosting seafood supply efficiency. However, it is equally vulnerable to conflicts over coastal resources and the environment. For instance, shrimp viruses once devastated China’s shrimp farming industry, while environmental pollution caused widespread scallop mortality in marine aquaculture [9]. Third, “restrictions outweigh deregulation.” Over the past four decades, China has implemented dozens of regulations enforcing strict fishing moratoriums to curb overfishing. These measures aim to break the vicious cycle of depleting resources and promote sustainable marine food production systems. Prior to the first summer fishing moratorium in 1995, fishery resources suffered prolonged, intensive overexploitation. Fourth, the proportion of intensive and modern marine aquaculture remains low, with intensive farming—represented by cage and indoor aquaculture—accounting for only 5.8% of total marine aquaculture production [6].

China’s “comprehensive food perspective” has ushered in a new cycle of marine food production, bringing a series of policy dividends. Driven by demand, the development of marine food production systems is now encountering significant opportunities. Aquaculture and fishing technologies have made substantial progress in recent years, particularly with the application of digital and information systems across production stages, which can significantly enhance overall efficiency [10]. However, marine food production systems currently face major challenges. First is the resource dilemma: encompassing production resources, human resources, and natural resources. Marine food production systems increasingly depend on the sophistication and reliability of equipment such as fishing vessels and gear, the technical proficiency of specialized practitioners, and the stability of resources and the environment [11]. Second is production pressure: Ample yields and substantial profits are key indicators for maintaining practitioners’ production motivation and scientific production attitudes, particularly under the backdrop of strictly enforced stringent regulatory policies [12]. Third are governance challenges. Marine food production systems confront both the practical difficulties of large enforcement areas and extended enforcement cycles, as well as emerging issues like the increasing concealment and organization of illegal fishing [13].

Sustainability assessments for marine resource development and utilization have become relatively well-established, but comprehensive evaluation studies focused on marine food production system development remain scarce [11]. This study aims to provide a comprehensive evaluation of the historical evolution and current status of China’s marine food production systems, analyze their temporal and spatial heterogeneity, and quantitatively identify key developmental obstacle factors. These findings will inform evidence-based policy recommendations for future development. The marginal contributions of this study are as follows: First, methodologically, it pioneers the introduction of text analysis methods in marine food production system evaluations, enhancing the objectivity and comprehensiveness of the evaluation indicator system. Second, theoretically, by constructing a goal-oriented, endogenously driven, and exogenously secured theoretical framework, it provides a three-dimensional framework for the indicator system encompassing benefits, resources, and governance, offering a new theoretical framework reference for research in this field. Third, in terms of analytical dimensions, the introduction of an obstacle degree model enables quantitative research on developmental barriers, expanding the analytical scope of marine food production systems beyond existing studies.

## 2. Literature Review and Theoretical Framework

### 2.1. Literature Review

#### 2.1.1. Conceptual Evolution of Marine Food Production Systems

The theoretical concept of China’s marine food production system was first proposed in 1996. Professor Bao Jianzhong advocated the concept of “blue agriculture,” conducting research on how to build a food supply system based on the food resources contained within marine ecosystems, defining it as humanity’s “second granary.” In 2008, Professor Tang Qisheng further refined the concept of the marine food production system, vigorously advocating the “Blue Ocean Food Initiative.” He proposed establishing a modern marine fisheries development system and a blue ocean food science and technology support system, giving rise to the concept of the “blue granary.” The goal was to enhance the yield and quality of marine aquatic products while safeguarding the marine ecological environment [6]. The concept and scope of China’s marine food production system have matured: it encompasses economic activities centered on marine biological resources, primarily conducted in coastal tidal flats and deep oceans. Utilizing modern technology and advanced facilities, it involves activities such as enhancement, aquaculture, fishing, deep processing, storage, and distribution to sustainably provide abundant, high-quality marine food for humanity. Marine aquatic product production based on marine ecosystems forms the core and foundation of marine food production system development. Its objectives include ensuring national food security, optimizing dietary structures, and promoting the healthy development of marine fisheries [14]. Globally, marine food production systems were historically overlooked in food system analysis and policy research due to biases favoring traditional agriculture. However, recent academic discourse increasingly recognizes marine food production systems as equally significant as terrestrial agricultural systems [15].

#### 2.1.2. The Strategic Significance of Marine Food Production Systems

The strategic significance of developing marine food production systems can be summarized in two key aspects: first, ensuring food supply and food security; second, improving the dietary structure and health levels of the population [16]. The finite carrying capacity of terrestrial resources struggles to meet the growing demand for animal-based foods. Tapping into the food supply potential of marine ecosystems alleviates pressure on terrestrial resources, plays a vital role in optimizing agricultural structures and enhancing resource utilization efficiency, and can also help mitigate the increasing food trade restrictions and volatility China currently faces [17]. Demand for protein-rich foods continues to climb annually. Land-based constraints limit the expansion of traditional animal protein sources like livestock farming and freshwater aquaculture, creating structural imbalances in food supply. Marine foods exhibit a pronounced substitution effect for terrestrial animal-based products, already contributing 12.56% of total animal-derived nutrients—a proportion poised to rise further as supply-side demands evolve [18]. From a health perspective, seafood contains abundant high-nutrient biological substances and is significantly lower in calories than terrestrial poultry and livestock meat products [18]. Furthermore, from an ocean economy standpoint, the production and trade within marine food production systems play a role in increasing consumer supply, creating employment opportunities, and promoting technological advancement [19].

#### 2.1.3. Development Potential of Marine Food Production Systems

The development potential of marine food production systems requires comprehensive assessment across multiple components—including coastal, offshore, aquaculture, and capture fisheries—using diverse methodologies. From a natural resource perspective, the production potential of coastal fisheries is determined by the net primary productivity of coastal fishery resources. According to the Ministry of Agriculture and Rural Affairs of China, the maximum sustainable yield for China’s coastal fisheries remains above 10 million tons. Regarding the productivity potential of distant-water fisheries, reports from the Food and Agriculture Organization of the United Nations (FAO) and related studies indicate that global marine fishery resources generally face overfishing and resource depletion. Consequently, the overall growth prospects for China’s distant-water fisheries production are not optimistic. Regarding marine aquaculture, since China’s Reform and Opening-up in 1978, the transition to a market economy propelled rapid development in marine farming. Marine aquaculture output surged from 4 million tons in 1985 to 25 million tons in 2000, achieving an average annual growth rate of 13% [20]. According to projections by the Food and Agriculture Organization of the United Nations (FAO), China’s demand for aquatic products will exceed 100 million tons by 2035. Overall, constrained by resource and environmental limitations, the future growth of China’s marine animal food production will be slow with limited expansion potential. From a production economics perspective, enhancing output per unit of input through technological progress represents a crucial pathway to improving future production potential. Offshore conservation, development of mid-to-upper water layers, expansion of polar fisheries, and the establishment of modern marine ranches with ecological restoration and resource enhancement functions are emerging as new directions for China’s sustainable green marine aquaculture development [6].

#### 2.1.4. Multidimensional Challenges Facing Marine Food Production Systems

On the supply side, marine capture fisheries production has experienced sustained negative growth, while the average annual growth rate of marine aquaculture production has slowed to approximately 5%. Overall, the compound annual growth rate of marine food supply remains below 2%, representing a significant decline from the 1980s [20]. At the environmental-ecological level, marine food production systems face compound pressures from human activities: oceans absorb approximately one-third of the carbon dioxide released into the atmosphere by humans. This continuous decline in pH alters the chemical composition of marine carbonates, leading to ocean acidification (OA). Since pre-industrial times, surface ocean pH has decreased by approximately 0.1 units—ten times faster than over the preceding 300 million years [21]. Through the food chain, these changes will ultimately exert substantial impacts on marine food quality [22]. Coastal waters have become increasingly polluted, severely damaging marine biodiversity and reducing fishery resources, with resource structures becoming simplified [23]. For a long time, the growth of China’s marine aquaculture industry has primarily relied on intensive inputs of capital, labor, and other factors. Coastal waters within 20 m of the isobath line and tidal flats have long maintained high-density, extensive farming practices, pushing the ecological carrying capacity of nearshore environments to near warning levels [7]. According to data from China’s environmental monitoring agencies, surveys of 760,000 hectares of key marine aquaculture zones nationwide revealed that 78.1% and 52.1% of monitored areas exceeded standards for inorganic nitrogen and active phosphate, respectively. On the governance side, challenges to the stable development of marine food production systems include illegal fishing, inefficient technologies, inadequate laws and regulations, and policy volatility [24].

### 2.2. Theoretical Framework

**Figure 1:**
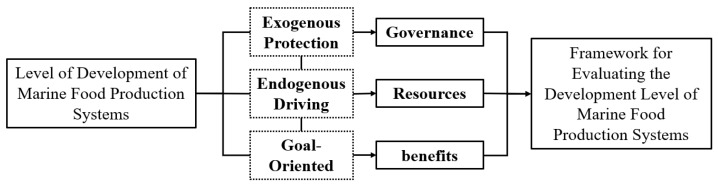
Research framework for the development level of marine food production systems.

A thorough and objective theoretical analysis of marine food production systems serves as a prerequisite for comprehensive evaluation and obstacle factors analysis. Even when employing relatively objective textual analysis methods to screen specific indicators within our research design, this process still requires a theoretical framework to delineate boundaries and establish hierarchies for indicator selection. Meeting this requirement necessitates establishing a well-structured theoretical framework with clearly defined boundaries, enabling a comprehensive deconstruction of the system’s components and internal logic [25]. From a systems theory perspective, marine food production systems encompass two primary dimensions: ecological environments and economic outputs. Governance and regulation form the foundational basis ensuring the operation of these two dimensions. Viewed through the lens of ecological-economic systems theory, marine food production systems can be summarized as comprising four subsystems: ecology, technology, production, and consumption [26,27]. Research by Martínez, Intralawan, Vázquez, Pérez-Maqueo, Sutton and Landgrave [28] emphasized that assessments of marine ecology and the marine economy should be conducted across three dimensions: ecological resources, economic benefits, and social governance. As illustrated in Figure 1, this study integrates these theoretical frameworks to organize key components of marine food production systems within a logical progression framework of “exogenous safeguard, endogenous drive, goal oriented”. It proposes a “benefits-resources-governance” research framework and constructs an evaluation indicator system based on this framework.

#### 2.2.1. Goal-Oriented: Benefits

Production volume and revenue serve as the core drivers of system development, addressing the questions of “how much to produce?” and “how much to earn?” In the context of China’s evolving ‘Greater Food’ approach, the fisheries sector has transcended its traditional role as a mere economic activity to become a strategic cornerstone of national food security and rural revitalization. It constitutes a complex socio-economic nexus that integrates fishing, aquaculture, processing, and cold chain logistics, these activities supply fishery products for human consumption and provide inputs for processing in other sectors, generating direct and indirect socioeconomic benefits [29]. Beyond its direct commercial value, the sector acts as a critical buffer for coastal employment and a driver of inclusive economic growth in underdeveloped maritime regions, thereby addressing the urban-rural development imbalance. China’s annual terrestrial food protein supply continues to grow at 2.83%, while the marine food production system’s protein supply increases by only 0.27% [30]. The core objective of marine food production system development is to alleviate pressure on terrestrial agricultural protein supply and reduce import dependency through marine food production. Maintaining and enhancing marine food yields is therefore imperative. Beyond production volume, profitability serves as a crucial indicator for sustaining marine food production system vitality. China’s marine fishery resource development primarily relies on individual aquaculture and fishing operations, characterized by a more dispersed and independent labor structure compared to terrestrial agriculture. Therefore, achieving expected returns is crucial for sustaining fishermen’s investment, maintaining scientific production practices, and preventing destructive competition [31]. Substantial returns can also attract more diverse stakeholders to participate in the system’s development.

#### 2.2.2. Endogenous Drive: Resources

The sustainable advancement of marine food production systems is now confronted with three interconnected socio-economic dilemmas: spatial competition, demographic shifts, and technological adaptation. The question of ‘Where to cultivate’ extends beyond assessing natural endowments; it involves navigating the complex trade-offs between marine spatial planning, biodiversity conservation, and industrial expansion. The question of ‘Who will cultivate’ reflects a pressing social reality—the aging workforce in coastal communities and the urgent need to attract younger, skilled labor through professionalizing the sector. Meanwhile, ‘How to cultivate’ demands a paradigm shift from resource-intensive practices to knowledge-intensive innovation, balancing productivity gains with environmental carrying capacity. The relationship between marine food production systems and natural resources similarly aligns with the triple role theory [32]. Marine food production systems are simultaneously victims of natural resource degradation, perpetrators of natural resource damage, and protectors mitigating environmental change. The accumulation of excrement from marine aquaculture organisms and nutrients from uneaten feed degrades the ecological environment of aquaculture areas, which in turn negatively impacts the growth of farmed marine organisms [33]. Natural resources thus determine the upper limit and stability of marine food production system development. Regarding human resources, the depletion of fishery resources poses significant challenges for resource users and policymakers. Marine food production systems also face the dilemma of losing effective labor while undergoing urbanization [34], making it easier for imbalances between laborers and technical personnel to emerge. Regarding production resources, the modernization of means and objects of labor remains inadequate, with relatively rudimentary technical equipment [35]. Enhancing technological efficiency can positively impact the overall endogenous driving forces.

#### 2.2.3. Exogenous Safeguard: Governance

Governance constitutes the critical exogenous safeguard for marine food production systems, raising two institutional questions: ‘Who governs?’ and ‘How?’ China’s marine food sector has long suffered from fragmented governance, where jurisdictional overlaps between fisheries, environmental, and maritime agencies create regulatory loopholes and collective action problems. This institutional fragmentation has perpetuated a productivist paradigm that prioritizes output expansion over ecological carrying capacity, failing to resolve the underlying socio-economic contradictions in resource allocation [36]. Most of China’s marine areas exhibit relatively low primary productivity and weaker resource endowments [37], posing greater challenges to balancing production output with regulatory control. The state has introduced a series of measures, such as the “dual control” policy limiting fishing vessel numbers and total engine power, along with supporting initiatives like “vessel reduction and industry transition.” China’s fisheries policy has transitioned from resource exploitation to ecological conservation [38] and shifted from input-based control management to output-based control management [31]. Key governance actors include fisheries enforcement agencies and corresponding fisheries management personnel. The density of management institutions and teams directly impacts governance effectiveness [39]. Governance methods emphasize both monitoring and intervention, combining fixed-point monitoring with proactive management. The effectiveness of monitoring and governance should be evaluated based on actual disaster losses incurred by the industry.

## 3. Methodology

### 3.1. Principles for Indicator Construction

Whether through theoretical framework analysis grounded in context and literature, indicator selection based on textual analysis, or subsequent refinement of specific indicators in conjunction with data sources, adherence to unified principles for indicator development is essential. This ensures consistency throughout the entire process—from framework construction to the confirmation of specific indicators [40]. First is the principle of comprehensiveness: although output serves as the primary outcome indicator for marine food production system development, pursuing output volume is by no means the sole objective. Variables underpinning stable production and supporting sustainable development constitute critical dimensions requiring consideration in indicator construction. Second is the principle of goal orientation: the ultimate purpose of marine food production system development is ensuring a stable and diverse supply of aquatic products amidst global supply chain uncertainties, while fundamentally improving the livelihoods and economic resilience of coastal populations. In the pursuit of common prosperity, it is crucial that the benefits are equitably distributed along the value chain—from small-scale fishing households to industrial enterprises—rather than merely increasing aggregate output. The construction of the indicator system must be directed towards these final outcomes, incorporating measurement elements at each key node. Third is the principle of operational feasibility: the functional purpose of constructing evaluation indicators is to assess the development level of China’s marine food production systems over multiple years. Specific indicators should be readily accessible, easily processed, and possess continuity.

### 3.2. Methodology for Indicator Selection

**Figure 2:**
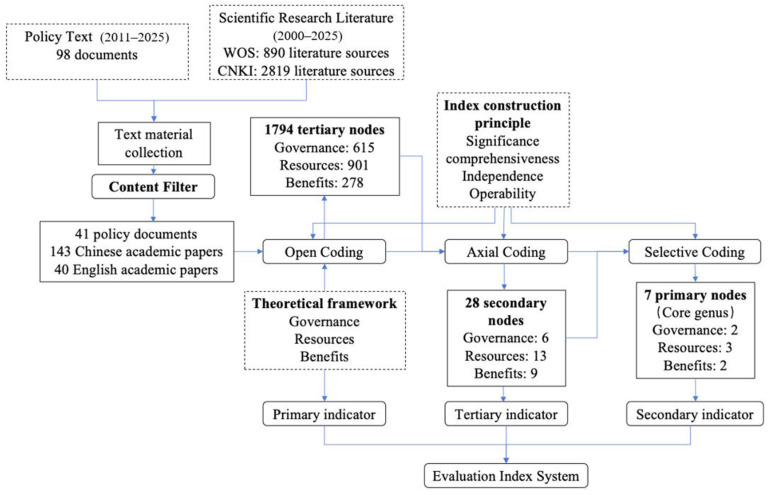
Indicator selection process.

In previous research on constructing indicator systems, the subjectivity inherent in indicator selection often undermines the authority of both the system itself and its evaluation outcomes. Adhering to the principle that “everything is data” [41], this study employs a combined approach of theoretical framework analysis and data-driven objective refinement. Firstly, the research context, literature review, and theoretical analysis delineate the fundamental structure and boundaries for the indicator system. Subsequently, key information segments are extracted from extensive policy documents and authoritative literature using text analysis methods to identify critical nodes at various levels aligned with the research objectives. Subsequently, specific indicators at each level were progressively confirmed based on the theoretical framework, text analysis outcomes, and indicator construction principles. Finally, objective weighting methods were applied to assign weights to each indicator, followed by comprehensive evaluation and multidimensional analysis. The overall process of constructing the indicator system is illustrated in Figure 2.

#### 3.2.1. Textual Analysis Methods and Primary Materials

Based on the grounded theory proposed by Glaser and Strauss [42], this study utilized policy documents and authoritative journal articles as primary reference texts. The scope of the search encompassed China National Knowledge Infrastructure (CNKI), Web of Science, and selected government official websites. Keywords centered on the development of marine food production systems were employed to accumulate textual materials for analysis. During the collation and refinement of literature, Nvivo15 software served as the principal analytical tool. Nvivo 15 is a mainstream analytical software capable of organizing and analyzing disorganized information, frequently employed in qualitative research. It enables in-depth processing and analysis of primary data such as texts and interview transcripts. Through a bottom-up inductive process across hierarchical nodes, it progressively extracts concepts, categories, main categories, and core categories pertaining to specific research subjects, thereby exploring the intrinsic attributes and essential properties of research concepts and variables. Regarding policy documents, this study compared the core tenets of marine food production systems with the main themes of policy documents. Consequently, 41 policy documents were selected from a total of 98 relevant documents for textual analysis. Concerning academic literature, the study retrieved 2819 journal articles published between 2000 and 2025 via the China National Knowledge Infrastructure (CNKI) database. Initial screening of titles and abstracts yielded 375 documents, which were subsequently reviewed in detail to identify 143 Chinese-language publications closely related to marine food production systems research. Using the same methodology, a Web of Science search yielded 40 articles from 890 relevant publications. Detailed information on policy documents, Chinese literature, and English literature is presented in Supplementary Materials, Section S1, Tables S1–S3.

#### 3.2.2. Open Coding

The 41 policy documents and 183 journal articles were imported into Nvivo 15 software. Concepts were extracted through word-by-word and paragraph-by-paragraph coding, with high-frequency thematic terms annotated to comprehensively present relevant indicators. Following annotation of all nodes, the internal logical consistency of each node was reviewed. Nodes failing to meet requirements were partially deleted, ultimately forming 1794 third-level nodes. Among these, governance, resources, and benefits comprised 615, 901, and 278 nodes respectively.

These nodes represent the foundational elements reflecting the intrinsic characteristics of the research subject at the lowest level of hierarchical subordination. This process involved meticulously sifting through collected textual materials word by word, line by line, and event by event to identify original statements pertinent to marine food production systems, tagging them accordingly, and ultimately abstracting several concepts and categories. Throughout this process, personal “biases” and preconceived research “opinions” must be discarded, along with subjective emotions and fixed mindsets. Data should be coded according to its inherent state [43]. Details of the open coding process are presented in Supplementary Materials, Section S2, Table S4.

#### 3.2.3. Axial Coding

Further synthesis of the 1794 tertiary nodes yielded 28 secondary nodes. Governance, resources, and benefits comprised 6, 13, and 9 nodes respectively. These nodes will serve as the basis for selecting specific tertiary indicators in subsequent research. During coding, content with consistent connotations was grouped under the same reference point based on textual similarity. For instance, expressions such as “fishing and aquaculture equipment, types, quantity, and technological level,” “improving the equipment level of fishing vessels,” and “construction of aquaculture facility equipment” are uniformly categorized as “Fishing Gear Value,” while descriptions including “ecological conditions of estuaries, bays, tidal wetlands, etc.,” “area of coastal waters with good water quality,” and “environmental pollution in bays caused by artificial aquaculture” are grouped under “environmental quality of coastal waters.” These secondary nodes occupy an intermediate tier within hierarchical relationships, representing an initial consolidation of previously fragmented foundational elements. The primary task of this process is to identify and establish connections between conceptual categories and domains, thereby expressing the organic relationships among data segments—including structural, causal, and temporal relationships—by establishing “axes” through categorization to forge these links.

#### 3.2.4. Selective Coding

The selective decoding process further distills highly generalized “core categories”, yielding seven core elements. Governance, resources, and benefits form two, three, and two primary nodes respectively. These primary nodes will serve as the basis for dividing secondary indicators within the subsequent research’s indicator system. Specifically, within the primary nodes: the governance dimension encompasses administrative management and environmental governance; the resources dimension includes natural resources, production resources, and human resources; and the benefits dimension comprises system output and system output value.

### 3.3. Indicator System Construction

#### 3.3.1. Indicator System

Based on text analysis screening, coding results and the theoretical framework, a comprehensive evaluation indicator system for the development level of China’s marine food production system has been constructed, comprising three tiers of indicators. The framework of the indicator system is grounded in the theoretical framework’s articulation of three dimensions: goal orientation, endogenous drivers, and exogenous safeguards. The theoretical framework corresponds one-to-one with the first-level indicators of the system, emphasizing the driving role of natural resources, human resources, and production resources as the foundational basis for development. It also underscores the necessity of maintaining goal orientation throughout the development process and implementing real-time adjustments through governance systems. This approach enables a precise assessment of the developmental foundations, current status, and prospects of marine food production systems in coastal regions. During the construction of the indicator system, textual analysis provided evidence-based support for the induction of secondary indicators and the selection of tertiary indicators. Adhering to the principles of comprehensiveness, goal-orientation, and operational feasibility, the textual analysis findings were translated into concrete, actionable indicators, culminating in the establishment of the indicator system for assessing the development level of China’s marine food production systems, as presented in Table 1. It should be noted that unlike pure regression analysis, correlations among indicators in multi-indicator comprehensive evaluation studies do not necessarily constitute methodological flaws. Moderate correlations often reflect intrinsic connections between different aspects within the same dimension. To further enhance the rigor of the research, we calculated Pearson correlation coefficients between all indicators. The results showed that the vast majority of correlation coefficients were below 0.8. For the few indicator pairs with correlations exceeding 0.8, the research team conducted a theoretical redundancy review based on grounded theory coding logic. Analysis revealed that while numerically correlated, these indicators conceptually represent distinct aspects of the same dimension. Detailed calculations and analysis of Pearson correlation coefficients are presented in Supplementary Materials, Section S2.

#### 3.3.2. Research Area and Data Sources

The research area of this study covers 11 coastal provinces and municipalities directly under the central government in China. Data sources for this study include the China Marine Economy Statistical Yearbook (2007–2022), China Fisheries Statistical Yearbook, China Statistical Yearbook, China Rural Statistical Yearbook, China Environmental Statistical Yearbook, and China Energy Statistical Yearbook. For a small number of missing indicator values, the latest publicly available data from the provincial (regional, municipal) departments of agriculture and rural affairs were used as the basis, supplemented by time series models and linear interpolation methods.

### 3.4. Measurement Methodology

#### 3.4.1. Calculation of Indicator System Weights

To overcome the shortcomings of subjective and objective weighting in comprehensive evaluations, this study references Tong, Xia, Sun, Yan, Li and Huang [32], combining the Analytic Hierarchy Process (AHP) with the Entropy Weight Method (EWM) to jointly determine the final weights for each indicator. Specifically, we employed the Fuzzy Analytic Hierarchy Process (FAHP) for subjective weighting. Fuzzy AHP resolves the consistency issue in judgment matrices while addressing convergence speed and accuracy, yielding ranking vectors more aligned with reality. The basic steps for FAHP calculation are as follows: First, decompose the numerous complex factors involved in the research question into objective, criterion, and indicator layers, forming a top-down hierarchical analysis structure. In this study, the objective layer, criterion layer, and indicator layer correspond to the first-level, second-level, and third-level indicators in Table 1, respectively. Second, based on the established hierarchical structure, conduct pairwise comparisons of the importance of each element within the same layer relative to a criterion in the preceding layer. To quantify these comparisons, this study employs a 0.1–0.9 scale method to construct a priority judgment matrix, as shown in Formula (1). Third, to ensure consistency in judgment, the priority judgment matrix must be transformed into a fuzzy consistent judgment matrix. The calculation steps are shown in Formulas (2)–(4). The resulting matrix satisfies fuzzy consistency and can be directly used for weight calculation. Fourth, the row and column normalization method is employed to calculate the subjective weights of each element, as shown in Formula (5).

(1) $$F={\left(f_{ij}\right)}_{n\times n}$$

where the elements $f_{ij}$ represents the importance of element i relative to  j, and satisfies the antisymmetric condition $f_{ji}=1-f_{ij}$.

(2) $$a_{i}=\sum_{k=1}^{n}f_{ik},$$

(3) $$a_{j}=\sum_{k=1}^{n}f_{jk}$$

(4) $$r_{ij}=\frac{a_{i}-a_{j}}{2\left(n-1\right)}+0.5$$

where $a_{i}$ denotes the sum of elements in each row, with i,j=1,2,…,n. $r_{ij}$ represents an element of the fuzzy consistency judgment matrix.

(5) $$\omega_{i,j^{*}}=\frac{\sum_{j=1}^{n}r_{ij}}{\sum_{k=1}^{n}\sum_{j=1}^{n}r_{kj}}$$

where $\;\omega_{i,j^{*}}$ denotes the subjective weight of each indicator.

Following dimensionless processing, we applied the Entropy Weighting Method for objective weighting. The entropy weight method is an objective weighting approach based on information theory. Its fundamental principle utilizes information entropy to measure the degree of disorder within a system, reflecting the amount of information carried by indicator data to derive objective weights. The steps for calculating weights using the entropy weight method are as follows:

First, construct the raw data matrix as shown in Formula (6). Second, to eliminate the effects of differing indicator units and positive/negative orientations, standardize the raw data. Apply Formula (7) for positive indicators and Formula (8) for negative indicators. Third, calculate the proportion of indicator values using Formula (9). Fourth, by computing the weight of the th indicator, ultimately obtain the objective weight vector of the entropy weight method, as shown in Formula (10).

(6) $$X={\left(x_{ij}\right)}_{m\times n}$$

where m represents the coastal province under evaluation, and n denotes the number of evaluation indicators. This constructs the raw data matrix, where $x_{ij}$ is the j-th indicator value for the i-th sample.

(7) $$z_{\mathrm{ij}+}=\frac{x_{\mathrm{ij}}-\min\left\{x_{j}\right\}}{\max\left\{x_{j}\right\}-\min\left\{x_{j}\right\}}$$

(8) $$z_{\mathrm{ij}-}=\frac{\max\left\{x_{j}\right\}-x_{\mathrm{ij}}}{\max\left\{x_{j}\right\}-\min\left\{x_{j}\right\}}$$

where $\max\left\{x_{j}\right\}$ and $\min\left\{x_{j}\right\}$ represent the maximum and minimum values of the j-th indicator, respectively, yielding the standardized matrix $Z={\left(z_{\mathrm{ij}}\right)}_{m\times n}$.

(9) $$p_{\mathrm{ij}}=\frac{z_{\mathrm{ij}}}{\sum_{i=1}^{m}z_{\mathrm{ij}}}$$

where $p_{\mathrm{ij}}$ represents the proportion of the i-th sample’s value within the j-th indicator.

(10) $$\omega_{i,j^{**}}=\frac{1-e_{j}}{\sum_{j=1}^{n}(1-e_{j})}$$

where $1-e_{j}$ is termed the information entropy redundancy or difference coefficient. A higher value indicates a greater indicator weight.

This study employed the Minimum Relative Information Principle [44] to integrate subjective and objective weights, with the calculation process as follows:

(11) $$\min F=\sum_{j=1}^{k}\omega_{i,j}(\ln\omega_{i,j}-\ln\omega_{i,j^{*}})+\sum_{j=1}^{k}\omega_{i,j}\left(\ln\omega_{i,j}-\ln\omega_{i,j^{**}}\right)$$

min F denotes the minimum relative information entropy, i represents the number of secondary indicators, j denotes the number of tertiary indicators, $\omega_{i,j}$ signifies the composite weight of each indicator after calculation, $\omega_{i,j^{*}}$ indicates the subjective weight during computation, and $\omega_{i,j^{**}}$ denotes the objective weight during computation.

(12) $$\omega_{i,j}=\frac{{\left(\omega_{i,j^{*}}-\omega_{i,j^{**}}\right)}^{\frac{1}{2}}}{\sum_{j=1}^{k}\omega_{j}{\left(\ln\omega_{i,j^{*}}-\omega_{i,j^{**}}\right)}^{\frac{1}{2}}}$$

By applying the Lagrange multiplier method to further optimize Equation (1), we obtain Equation (2). It can be seen that when the geometric mean of the subjective weight $\omega_{i,j^{*}}$ and the objective weight $\omega_{i,j^{**}}$ is taken, the information content is minimized.

#### 3.4.2. Regional Obstacle Factors Calculation Model

To further explore developmental shortcomings in marine food production systems across different regions, this paper adopts the obstacle factors model methodology employed by Zhao, Fang, Liu and Zhang [45] and builds upon the practices of Qiao, Fan and Yin [25] in the field of China’s fishery economic resilience, this study introduces the obstacle factors model to measure the degree of obstruction caused by indicators across different dimensions to the development of marine food production systems. This approach identifies key obstacle factors to inform policy formulation. The obstacle factors model has been applied with relative maturity in measuring dimensions such as fishery modernization, sustainable agricultural development, and high-quality agricultural development. To ensure research consistency and coherence, the obstacle factors model employs the same weighting as that used for measuring the development level of marine food production systems. Based on the Min-max normalization applied earlier, the standardized values of all indicators fall within the range [0, 1]. Within this framework, the value 1 represents the ideal state or target value for each indicator—that is, the optimal level observed across all samples during the study period. Thus, the actual measurement of $\left(1-N_{ij}\right)$ captures the gap between each indicator’s actual performance and its ideal benchmark, reflecting the degree of deviation from the optimal level. The calculation process is as follows:

(13) $$O_{i,j}=\frac{\left(1-N_{i,j}\right)\times \omega_{i,j}}{\sum_{j=1}^{33}\left[\left(1-N_{i,j}\right)\times \omega_{i,j}\right]}$$

(14) $$O_{i}=\sum_{j=1}^{n_{j}}O_{i,j}$$

In the formula, $O_{i,j}$ denotes the obstacle degree of the j-th single indicator under the i-th criterion layer to the development of the marine food system; $N_{i,j}$ is the standardized value of the indicator (based on Min-max normalization, where 1 represents the optimal value); $\omega_{i,j}$ is the composite weight of the indicator; and $O_{i}$ represents the degree of obstruction to regional development for the i-th criterion layer.

It is particularly noteworthy that in applying the FAHP and EWMs, we adopted row normalization and range normalization respectively. This design stems primarily from the following considerations. First, traditional AHP typically employs the characteristic root method to solve weights, which requires complex iterative calculations and must pass consistency tests. FAHP, however, constructs a fuzzy consistency matrix that inherently satisfies consistency conditions, eliminating the need for such tests. Under this premise, row-sum normalization offers computational simplicity and information preservation. Second, the normalization method in the EWM must simultaneously eliminate dimensions while preserving data variability. The range normalization method imposes no assumptions on data distribution, avoids producing varying compression ratios, and aligns with the ideal benchmark setting for the impedance model. Therefore, it is superior to other normalization methods such as Z-scores.

## 4. Results

### 4.1. Analysis of China’s Marine Food Production System Development Level Results

**Figure 3:**
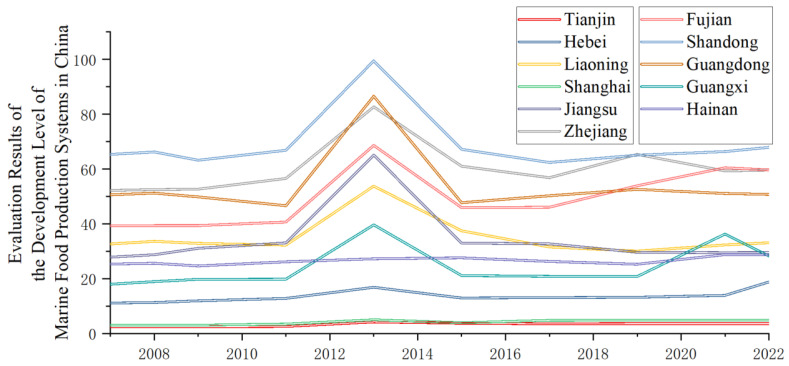
Analysis of regional heterogeneity in the level of development of marine food production systems.

Table 2 and Figure 3 present the development level ratings for China’s marine food production system. Overall trends indicate that the national marine food production system’s development can be divided into three phases: an expansion period, an adjustment period, and a differentiation period. The expansion period spanned 2009–2013, during which coastal regions experienced rapid growth in marine food production system development following the introduction of a series of favorable national policies. Subsequently, from 2015 to 2019, the system entered an adjustment phase. On one hand, the state-imposed restrictions on production models through measures such as establishing marine ecological red lines and strengthening fisheries and fishing vessel controls to address ecological and environmental concerns and promote scientific production. On the other hand, the stagnation during this adjustment period was also a consequence of the unrestrained growth during the expansion phase. From 2019 to 2022, the national marine food production system entered a period of differentiation, which can be divided into four tiers based on overall development levels. The first tier comprises Shandong and Zhejiang provinces, which leverage their advantages in full industrial chains and leading technologies, achieving average scores above 59 points. The second tier includes Fujian and Guangdong, regions dominated by export-oriented economies, with average scores ranging from 50 to 59 points. The third tier comprises Liaoning and Jiangsu, where development has stagnated or even regressed due to slow industrial transformation and resource degradation, with average scores between 29 and 34 points. The fourth tier includes Hebei, Guangxi, and other regions, averaging below 24 points. Weak resource foundations and high policy dependency are the primary reasons for their below-average development levels.

Fluctuations in development levels also stem from significant structural causes. From a governance perspective, formulating policies to bridge the gap between human needs and environmental degradation represents the most crucial and effective pathway [46]. From the macro perspective of sustainable marine economic development, unlocking potential while maintaining sustainability hinges on three critical indicators: responding to environmental change, ensuring food security, and preserving the stable functioning of biological ecosystems [47]. Considering real human needs, adjusting industrial structures through market mechanisms is a vital means to achieve supply-demand balance while accounting for ecological carrying capacity [48]. We categorize these into three types: policy-driven, market-driven, and environment-driven. First, the dominant structural cause in China’s marine food production system development is policy-driven fluctuations. A series of policies, exemplified by the Regional Comprehensive Economic Partnership Agreement, have driven demand growth in the marine food production system, enhancing development levels in regions like Fujian and Guangxi at the industrial vitality level. Additionally, in 2024, China’s central government outlined plans to accelerate deep-sea aquaculture development, establishing marine ranches and blue granaries. Although this falls outside our study’s timeframe, it undeniably represents the culmination of prior policy interventions. Market-driven fluctuations in development levels can be further categorized by their impact on overall supply-demand dynamics through trade and consumption: On the trade front, factors like the U.S. anti-dumping investigation on shrimp products during 2019–2021 caused a sharp decline in total exports, affecting the comprehensive scores of multiple regions that year. On the consumption side, surges like the explosive demand for ready-to-eat seafood dishes in Zhejiang significantly boosted the development and output value of marine food-related industries within the region. Environment-driven fluctuations primarily refer to those caused by restrictive measures implemented to protect the ecological environment of production areas. Although these fluctuations often yield negative short-term outcomes, policy adjustments are typically aimed at achieving sustainable and healthy development. Over longer timeframes, environment-driven fluctuations tend to be positive. For instance, between 2021 and 2022, benefiting from the dividends released by years of environmental policy protection, shellfish production in Hebei Province rebounded by 32%.

### 4.2. Chronological Obstacles to the Development of the National Marine Food Production System

The analysis results of obstacle factors to the national marine food production system are presented in Table 3. Examining the temporal characteristics of the proportion of obstacle factors reveals three primary phases in their evolution. The first phase was dominated by human resource constraints, with the proportion of human resource obstacle factor steadily increasing to 42.48% by 2015. This period featured dual pressures of labor shortages and lagging labor quality, most notably the aging of aquaculture households where workers over 50 accounted for over 60% of the workforce, alongside significant shortages of offshore crew members. The second phase was dominated by production resource constraints, with their share reaching 47.61% in 2013. The primary factor during this period was the slow development of production tools. Notably, the periods dominated by production resource constraints and human resource constraints overlapped significantly, indicating that China’s marine food production system was persistently constrained by resource shortages from 2008 to 2015 before gradually improving. Third is the period from 2017 to 2020, dominated by system output constraints. Output was constrained both by restrictive policies such as fishing bans and as a long-term consequence of resource limitations, further exposing the impact of lagging resource development on the overall system’s advancement. After experiencing fluctuations, the degree of human resource constraints has shown an upward trend in recent years, further highlighting the critical role and practical challenges of human resources in the development of labor-intensive production systems. Additionally, the degrees of constraints related to natural resources, system output value, environmental governance, and administrative management have all gradually decreased amid fluctuations, indicating that the effects of natural resource conservation, industrial upgrading, and governance improvements are gradually becoming evident.

### 4.3. Analysis of Current Obstacles to Marine Food Production System in Coastal Regions

**Figure 4:**
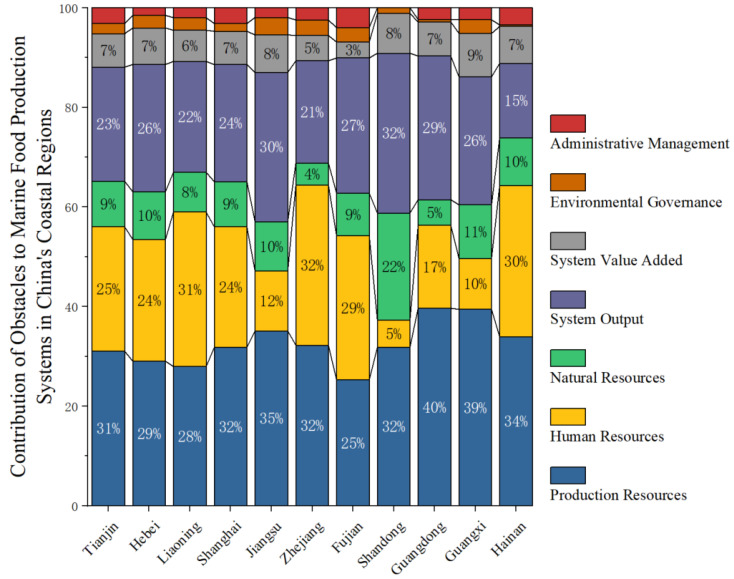
Analysis of regional heterogeneity in the degree of obstacle factors to the development of marine food production systems (2022).

The 2022 regional heterogeneity analysis results are shown in Figure 4, revealing the regional differentiation characteristics of core obstacles. Production resource constraints exhibit a north–south divergence, with higher levels in the south and lower levels in the north. Guangdong, Guangxi, Jiangsu, and Hainan provinces have the highest production resource constraint levels. The weak foundation of equipment manufacturing may be the cause of high production resource dependency and significant constraints in these regions. Human resource constraints are most pronounced in Zhejiang, Liaoning, and Hainan, reflecting three distinct patterns affecting marine food production system development: declining agricultural labor force in economically advanced regions, agricultural population outflow in areas with net population loss, and insufficient agricultural labor in regions with weak population bases. System output constraints showed relatively balanced levels, with Shandong, Jiangsu, and Hebei exhibiting higher constraints. This reflects two factors: first, constraint proportions correlate strongly with natural resource depletion—as evidenced by Shandong and Jiangsu’s concurrently high natural resource constraints. Second, regions achieving high output yet maintaining substantial constraint proportions indicate risks of underdeveloped production potential and structural imbalances, cautioning against prioritizing output alone.

## 5. Conclusions and Implications

### 5.1. Research Findings

China’s marine food production system is characterized by “differentiated development, painful transition, and ecological priority.” During the expansion phase from 2009 to 2015, driven by policy support, the comprehensive development level of China’s marine food production system saw significant leaps in most regions, though it also harbored risks of resource depletion. Through the establishment of ecological red lines and regulatory policy adjustments, the system entered a period of adjustment from 2015 to 2019. Subsequently, from 2019 to 2020, the system exhibited pronounced differentiation. In terms of driving factors: favorable policies can stimulate short-term growth but are unsustainable; market forces can propel industrial transformation but also amplify volatility; environmental and governance drivers can achieve long-term sustainable development through short-term pain.

Development obstacle factors in China’s marine food production system primarily manifest across three dimensions: human resources, production resources, and overall capacity. These obstructive factors exhibit pronounced regional differentiation and have dominated systemic stagnation at different developmental stages. Human resource constraints dominated systemic obstacles from 2008 to 2015; production resource constraints dominated from 2013 to 2017, showing a pattern of higher intensity in the south and lower in the north; System output has been the primary constraint since 2018, significantly impacting Shandong and Jiangsu. The temporal patterns of these constraints reveal a potential mechanism chain: human resource shortages lead to reduced production capacity, which further entrenches extensive production practices, thereby increasing governance challenges. This ultimately results in insufficient production resource supply, negatively impacting output and ecological restoration while diminishing governance effectiveness—creating a risk of vicious cycles.

### 5.2. Policy Recommendations

#### 5.2.1. Establish Carrying Capacity-Based Spatial Zoning with Dynamic Adjustment Mechanisms

Translate ecological limits into enforceable management tools. First, delineate marine spatial planning into permitted, restricted, and prohibited zones for fisheries, with differentiated discharge permits and aquaculture capacity licensing. Second, reform dual-control enforcement by introducing seasonal catch quotas and community-based co-management to mitigate rebound effects from blanket moratoriums. Third, address nutrient pollution and tidal flat degradation through cross-sectoral ecological compensation funds, requiring benefiting industries to finance restoration rather than relying solely on fiscal appropriations.

#### 5.2.2. Professionalize the Maritime Workforce Through Income Stabilization and Skill Certification

Address labor shortages from both income security and career attractiveness. In the short term, during fishing moratoriums, local governments should facilitate flexible employment platforms connecting fishers with seasonal labor demand in offshore wind, coastal tourism, and seafood processing to ensure income continuity. Simultaneously, extend social insurance or occupational injury coverage to fishers under urban employee schemes. Leverage modern marine ranching and deep-sea aquaculture vessels to create certified vocational roles developed through industry-academia partnerships, repositioning marine fisheries as a skilled, career-track profession for younger generations.

#### 5.2.3. Establish a Multi-Scale Diagnostic Assessment Mechanism with an Annual Review Cycle

To translate assessment outcomes into actionable policy adjustments: First, implement differentiated weighting based on coastal cities’ developmental stages. Second, mandate annual assessments rather than periodic reviews to capture rapidly evolving ecological and market dynamics, establishing early warning systems to identify bottlenecks before they escalate. Third, institutionalize a closed-loop: for high-output, high-constraint regions like Shandong, initiate targeted audits and reallocate support resources to identify weak areas instead of implementing universal subsidies. Finally, integrate this system into provincial performance evaluation mechanisms to ensure local governments implement diagnostic outcomes, transforming assessments from academic exercises into binding, refined local governance tools.

#### 5.2.4. Strengthen Productive Capacity Through Technological Upgrading and Efficiency-Oriented Incentives

Address the dual challenge of production resource constraints and the “high output, high obstacle” trap revealed by the findings. First, tackle persistent equipment and infrastructure bottlenecks by establishing targeted modernization programs for fishing fleets, aquaculture automation, and cold chain logistics in regions where weak manufacturing foundations constrain productivity. Second, reform production incentives by coupling support measures with resource efficiency audits: channel subsidies toward yield increases achieved through technological innovation rather than resource intensification, thereby breaking the cycle where high output coexists with structural vulnerabilities. Third, leverage demonstrated advances in modern marine ranching and deep-sea aquaculture by creating cross-regional technology transfer platforms, enabling lagging areas to leapfrog through adoption of proven practices. Finally, institutionalize regular production technology assessments to identify emerging bottlenecks before they escalate into systemic constraints, ensuring capacity building keeps pace with evolving demand while maintaining alignment with ecological carrying capacity.

### 5.3. Research Limitations and Future Directions

This study has several limitations that warrant further exploration in future research. First, the interactions among drivers causing fluctuations in marine food production systems remain understudied, future work should consider econometric methods such as PVAR models to quantify the impacts of different factors. Second, while the analysis of barriers revealed a hypothetical chain of mechanisms—from human resource shortages to production constraints to final output limitations—the causal pathways require further validation using microdata. Third, the comprehensiveness of the indicators covered in this study is limited. Certain factors that are difficult to quantify could not be included due to unavailable data, which may affect the completeness of the evaluation results. Additionally, the endogenous relationships among indicators are difficult to fully disentangle. While correlation coefficient tests indicate no severe multicollinearity, some indicators within the “Production Resources” and “System Output” dimensions still exhibit strong economic logical correlations. Fourth, while regional heterogeneity analysis captures spatial variations, it fails to fully account for inter-regional linkages that propagate constraints across administrative boundaries. Subsequent studies may employ network analysis to model these interdependencies. Finally, this study’s timeframe ends in 2022, precluding assessment of recent deep-sea aquaculture initiatives and post-pandemic recovery patterns. Extending the research period in future work will be crucial for evaluating whether sustainable governance transitions yield enduring improvements.

## Figures and Tables

**Table 1:** Indicator system for evaluating the development level of China’s marine food production system.

Primary Indicator	Secondary Indicator	Tertiary Indicator & Unit	Indicator Weight	Indicator Attribute
Resources	Production Resources	Number of Motorized Fishing Vessels (vessels)	2.65%	Positive
		Motorized Fishing Vessel Power (kW)	2.35%	Positive
		Fishing Gear Value (10,000 yuan)	8.16%	Positive
		Marine Aquaculture Area (ha)	4.70%	Positive
		Number of Fishing Ports	5.76%	Positive
		Number of Aquatic Product Processing Enterprises	3.23%	Positive
		Number of Aquatic Product Cold Storage Facilities	3.52%	Positive
	Human Resources	Fisheries-related population (persons)	3.18%	Positive
		Professional Personnel (persons)	2.33%	Positive
		Aquatic Technology Extension Personnel (persons)	3.92%	Positive
	Natural Resources	hallow Coastal Tidal Flat and Bay Cultivable Area (hectares)	3.34%	Positive
		Coastline Index (Continental Coastline Length/Total National Continental Coastline Length)	3.65%	Positive
		Island Index (Number of Islands/Total National Islands)	2.94%	Positive
Benefits	System Output	Marine Fish Production (tons)	3.12%	Positive
		Marine Shrimp and Crab Production (tons)	3.67%	Positive
		Marine Shellfish Production (tons)	3.30%	Positive
		Marine Algae Production (tons)	5.71%	Positive
		Other Marine Product Production (tons)	5.97%	Positive
		Marine Cephalopod Production (tons)	3.66%	Positive
	System Output Value	Marine Fishing Output Value (10,000 yuan)	2.56%	Positive
		Marine aquaculture output value (10,000 yuan)	3.66%	Positive
		Per capita net income of fishermen (10,000 yuan) Per capita net income of fishermen (10,000 yuan)	2.27%	Positive
Governance	Environmental governance	Number of coastal marine environmental quality monitoring points	3.01%	Positive
		Economic losses from fishery disasters (10,000 yuan)	2.10%	Negative
		Red Tide Disasters (hectares)	2.09%	Negative
		Land-Based Pollutants Discharged into Sea (tons)	3.07%	Negative
	Administrative Management	Number of Fisheries Law Enforcement Agencies	3.54%	Positive
		Number of Fisheries Management Personnel	2.54%	Positive

**Table 2:** Evaluation results of the development level of marine food production systems.

	2007	2010	2012	2014	2016	2018	2020	2022	Annual Growth Rate
Tianjin	2.49	2.50	2.89	3.45	3.54	3.45	3.50	3.58	3.19%
Hebei	11.10	11.87	13.04	12.75	13.42	13.07	13.52	14.77	6.35%
Liaoning	32.60	35.13	35.21	35.18	38.06	29.77	33.80	34.87	1.55%
Shanghai	3.00	3.08	3.78	4.10	4.46	4.88	4.93	4.93	3.63%
Jiangsu	27.85	32.45	30.82	33.44	39.13	31.36	30.69	29.02	4.07%
Zhejiang	52.16	55.09	59.73	61.15	60.62	61.13	59.91	59.42	1.64%
Fujian	39.30	39.51	43.67	43.14	48.50	50.66	56.49	60.74	4.22%
Shandong	65.33	65.61	60.82	65.74	67.83	64.75	67.32	67.57	1.85%
Guangdong	50.56	45.74	44.52	49.54	50.78	54.03	50.46	51.35	2.71%
Guangxi	17.99	21.19	20.75	20.97	21.73	21.40	21.59	19.92	5.42%
Hainan	25.30	25.24	26.65	25.00	27.18	24.44	28.48	28.53	0.89%

**Table 3:** National chronological obstacle factors levels for marine food production system.

Year	Production Resources	Human Resources	Natural Resources	System Output	System Value Added	Environmental Governance	Administrative Management
2007	34.19%	36.54%	3.47%	12.13%	10.72%	0.61%	2.34%
2008	35.53%	36.34%	3.31%	10.75%	11.31%	0.38%	2.38%
2009	34.19%	36.54%	3.47%	12.13%	10.72%	0.61%	2.34%
2010	32.59%	37.98%	3.62%	12.83%	10.34%	0.21%	2.43%
2011	30.16%	39.66%	1.71%	13.23%	11.53%	1.53%	2.18%
2012	33.14%	37.99%	1.98%	14.21%	7.98%	2.68%	2.02%
2013	47.61%	6.93%	3.26%	20.89%	11.31%	4.60%	5.41%
2014	32.16%	40.99%	2.25%	13.08%	7.41%	3.07%	1.03%
2015	36.74%	42.48%	2.32%	9.21%	6.37%	2.02%	0.86%
2016	37.24%	41.32%	2.13%	11.48%	6.47%	1.18%	0.18%
2017	32.08%	37.20%	1.50%	21.58%	4.72%	2.45%	0.48%
2018	29.93%	37.07%	0.17%	25.92%	3.60%	2.11%	1.20%
2019	26.12%	37.23%	0.27%	28.22%	3.68%	3.29%	1.20%
2020	27.86%	37.30%	0.27%	29.16%	2.96%	1.33%	1.12%
2021	21.54%	40.82%	0.00%	33.15%	2.36%	1.30%	0.83%
2022	28.96%	37.52%	0.11%	30.21%	0.80%	1.36%	1.06%
Average	32.50%	36.49%	1.86%	18.64%	7.02%	1.79%	1.69%

## Data Availability

The original contributions presented in this study are included in the article/Supplementary Materials. Further inquiries can be directed to the corresponding author.

## Supplementary Materials

The following supporting information can be downloaded at: https://www.mdpi.com/article/10.3390/foods15061031/s1, Table S1. Policy documents referenced for text analysis; Table S2. All Chinese literature references for text analysis; Table S3. All English literature references for text analysis; Table S4. Pearson Correlation Coefficients for Selected Tertiary Indicator Pairs.
