# Supplementary material for: Development Level and Obstacle Factors of China’s Marine Food Production System
Source: Foods. 2026 Mar 16;15(6):1031. doi: 10.3390/foods15061031 (PMC13025813; doi:10.3390/foods15061031)
Supplement: Supplementary file 1 [file foods-15-01031-s001.zip › foods-4158097-supplementary.pdf]

foods

MDPI

Supplementary Materials

Development Level and Obstacle Factors of China's Marine Food Production System

Haotian Tong, Xiaoting Zhang, Enjun Xia, Cong Sun and Jieping Huang*

School of Management, Beijing Institute of Technology, Beijing 100081, China
* Correspondence: cindy@bit.edu.cn; Tel.: +86-010-68918986

Supplementary Materials Section S1

Table S1. Policy documents referenced for text analysis.

No.	Document Title	Official Source	Release Date
1	Fisheries Law of the People's Republic of China (Latest Revised Edition)	Standing Committee of the National People's Congress	2026 (to be implemented)
2	Regulations on the Administration of Fishing Licenses	Ministry of Agriculture and Rural Affairs	2014
3	China Marine Disaster Bulletin	Ministry of Natural Resources	2023
4	Opinions on Promoting the High-quality Development of the Offshore Fisheries during the 14th Five-Year Plan Period	Ministry of Agriculture and Rural Affairs	2022
5	Marine Environmental Protection Law of the People's Republic of China	Standing Committee of the National People's Congress	2023
6	National Fisheries Development Plan for the 14th Five-Year Plan Period	Ministry of Agriculture and Rural Affairs	2021
7	Implementation Opinions of the CPC Liaoning Provincial Committee and the People's Government of Liaoning Province on Comprehensively Promoting the Construction of a Beautiful Liaoning	Liaoning Provincial People's Government	2024
8	Several Policy Measures of Liaoning Province to Consolidate and Enhance Economic Growth and Promote Continuous Recovery and Improvement	Liaoning Provincial People's Government	2023
9	Liaoning Province's 14th Five-Year Plan for Marine Economic Development	Liaoning Provincial People's Government	2022
10	Notice of the General Office of the People's Government of Hebei Province on Issuing Several Measures for Further Strengthening the Safety Production Management of Marine Fisheries	People's Government of Hebei Province	2023
11	National Coastal Fishing Port Construction Plan (2018-2025)	National Development and Reform Commission, Ministry of Agriculture and Rural Affairs	2018
12	Circular of the General Office of the People's Government of Hebei Province on Issuing the Work Plan for Re-inspection and Rectification of River and Sea Discharge in Hebei Province	People's Government of Hebei Province	2022
13	Notice of Hebei Provincial People's Government on Printing and Distributing the 14th Five-Year Plan for Ecological and Environmental Protection in Hebei Province	People's Government of Hebei Province	2022

14	Tianjin's 14th Five-Year Plan for Marine Economic Development	Ministry of Agriculture and Rural Affairs	2021
15	The 13th Five-Year Plan for National Fisheries Development	Ministry of Agriculture and Rural Affairs	2016
16	Work Specifications for the Compilation of Aquaculture Water Area and Beach Planning and Compilation Outline	Ministry of Agriculture and Rural Affairs	2016
17	Opinions of the Jiangsu Provincial Government on Strengthening the Prevention and Control of Pollution in Coastal Waters	Jiangsu Provincial People's Government	2015
18	The Approval of Jiangsu Provincial Government on the Marine Ecological Red Line Protection Plan of Jiangsu Province (2016-2020)	Jiangsu Provincial People's Government	2017
19	Opinions of the Jiangsu Provincial Government on Promoting the Construction of Modern Fisheries	Jiangsu Provincial People's Government	2014
20	Notice of Shanghai Ocean Bureau on Printing and Issuing Shanghai Ocean 14th Five-Year Plan	Shanghai Municipal People's Government	2021
21	Notice of Shanghai Ocean Bureau on Printing and Issuing Shanghai Ocean Management Work Summary in 2024 and Shanghai Ocean Management Work Points in 2025	Shanghai Municipal People's Government	2025
22	Shanghai Municipal People's Government	Jiangsu Provincial People's Government	2021
23	The State Council's Several Opinions on Promoting the Sustainable and Healthy Development of Marine Fisheries	the State Council	2013
24	Several Opinions of Zhejiang Provincial Bureau of Ocean and Fisheries on Strengthening the Control of Fishing Vessels and Implementing Total Management of Marine Fishery Resources	Zhejiang Provincial People's Government	2021
25	Zhejiang Province Marine Functional Zoning	Zhejiang Provincial People's Government	2018
26	The 13th Five-Year Plan for the Transformation and Upgrading of the Fisheries Industry in Zhejiang Province	Zhejiang Provincial People's Government	2016
27	Notice of the General Office of Zhejiang Provincial People's Government on Printing and Distributing the 13th Five-Year Plan for the Development of Marine Ports in Zhejiang Province	Zhejiang Provincial People's Government	2016
28	Opinions on Upholding and Improving the Basic Management System of Fisheries	Ministry of Agriculture and Rural Affairs	2012
29	The 12th Five-Year Plan for National Fishery Development (2011–2015)	Ministry of Agriculture and Rural Affairs	2011
30	Notice on Issuing the Implementation Plan for the "Five Major Initiatives" on Promoting Green and Healthy Aquaculture Technology	Office of the Ministry of Agriculture and Rural Affairs	2021
31	Fujian Province's 14th Five-Year Plan for Building a Strong Marine Province	Fujian Provincial People's Government	2021
32	Notice of Fujian Provincial People's Government on Printing and Distributing Fujian Marine Environmental Protection Plan (2011-2020) (Special Plan)	Fujian Provincial People's Government	2011
33	Opinions of Fujian Provincial People's Government on Building a Comprehensive Protection and Governance Framework from Mountain Top to Ocean	Fujian Provincial People's Government	2025
34	Opinions of Guangdong Provincial People's Government on Promoting the Transformation and Upgrading of Marine Fisheries and Improving the Development Level of Marine Fisheries	People's Government of Guangdong Province	2013

https://doi.org/10.3390/xxxxx

35	Notice of the Ministry of Agriculture and Rural Affairs on Further Strengthening the Control of Domestic Fishing Vessels and Implementing the Total Management of Marine Fishery Resources	Ministry of Agriculture and Rural Affairs	2015
36	Notice of the People's Government of Guangxi Zhuang Autonomous Region on Issuing the Guangxi Marine Industry Development Plan	People's Government of Guangxi Zhuang Autonomous Region	2010
37	Hainan Province Marine Economy Development Plan	Hainan Provincial People's Government	2006
38	Notice of the State Council on Issuing the National Marine Main Functional Zone Planning	the State Council	2015
39	Circular of the Ministry of Agriculture and Rural Affairs on Further Strengthening the Management of Fishing Vessels and Controlling the Intensity of Marine Fishing during the 12th Five-Year Plan Period	Ministry of Agriculture and Rural Affairs	2011
40	National Offshore Fisheries Development Plan for the 13th Five-Year Plan Period	Fishery and Fishery Administration Bureau, Ministry of Agriculture and Rural Affairs	2017
41	China's Development of Long-Distance Fisheries	Ministry of Agriculture and Rural Affairs	2023

Table S2. All Chinese literature references for text analysis.

No.	Title	Journal	Publication date (year-month)
1	Green Shipbuilding Concept of China Shipbuilding Enterprises	Ship Engineering	2010-09
2	China Coastal Provinces Blue Carbon Status and Capacity Assessment	Resource Science	2022-06
3	Measurement and Evaluation of Carbon Emission Efficiency in Marine Fisheries of China Coastal Cities	Research on Science and Technology Management	2015-08
4	China's Blue Granary Industry: Current Status, Issues and Countermeasures	Hubei Agricultural Science	2023-09
5	Calculation and Potential Assessment of 'Removable Carbon Sink' from Shellfish and Algae Cultivation in China's Offshore Waters	Journal of Applied Oceanography	2025-05
6	Random Frontier Analysis of China's Offshore Fishing Industry Production—Based on Provincial Panel Data	Agricultural Technology and Economics	2014-08
7	Analysis of Carbon Sequestration and Environmental Benefits of China's Seaweed Farming	China Journal of Ecological Agriculture	2014-09
8	China's Marine Strategic Emerging Industries Technology Innovation Collaborative Network Structure and Driving Mechanism	Management of Research and Development	2024-12
9	China Marine Fisheries Carbon Neutrality Capacity Assessment	China Agricultural Science and Technology Herald	2022-11
10	Research on the Carbon Balance Status and Influencing Factors of China's Marine Fisheries	Marine Science	2023-09

11	Spatiotemporal Evolution of Green Development Efficiency in China's Marine Fisheries—Based on an Uncertainty EBM Model	Resource Science	2025-05
12	Measurement and Temporal-Spatial Evolution of High-Quality Development in China's Marine Fisheries	Ocean Bulletin	2024-12
13	Measurement of Green Efficiency of China's Marine Carbon Sink Fisheries and Its Spatial Spillover Effects	China Rural Economy	2010-09
14	China Marine Industry Blue Carbon Source and Sink Identification and Carbon Sink Development Potential Preliminary Exploration	China Marine Economy	2024-10
15	Evaluation of the Effectiveness of the 'Dual Control' System for China's Marine Fishing Vessels and Its Implementation Adjustment	Fujian Forum	2016-11
16	Analysis of the Coordination Degree between China's Marine Aquaculture and Marine Ecological Environment	China Rural Economy	2013-11
17	Analysis of Coupled and Coordinated Development of Resources-Environment-Economy System in China's Marine Aquaculture Industry	Journal of Shanghai Ocean University	2022-09
18	Green Development Evaluation and Temporal-Spatial Evolution Characteristics of China's Marine Aquaculture Industry	geoscience	2013-10
19	Research on Green Development Evaluation of China's Marine Aquaculture Industry	China Agricultural Science and Technology Herald	2021-06
20	Spatial Evolution and Spillover Effects of Net Carbon Sink Efficiency in China's Marine Aquaculture	Marine Development and Management	2023-02
21	Research on Carbon Sink Assessment and Trading Mechanism of Marine Aquaculture Shellfish in China	China Agricultural Resources and Regionalization	2024-08
22	China's' Blue Granary' Production Potential Assessment Research	Rural Economy	2018-06
23	The Intrinsic Logic of the Transformation of China's Marine Environmental Governance Mode under the Overall System View	Academic Exchange	2025-03
24	Assessment of Marine Carbon Sink Resources and Potential in Zhejiang Province	Marine Development and Management	2023-12
25	Assessment of Carbon Sink Capacity of Major Shellfish and Algae in Zhejiang Province	Anhui Agricultural Science	2024-08
26	Path Optimization Analysis of Environmental Pollution Coordinated Governance in the Coastal Waters of the Yangtze River Delta	Marine and Lake Bulletin (Chinese and English)	2025-04
27	Estimation Method of Energy Consumption and Carbon Emission of Fishing Vessels and Research Status of Energy Conservation and Emission Reduction	marine fishery	2025-09
28	Evaluating the Coordinated Development of Ecological Security and Tourism Economy in the Coastal Waters of Pingtan Island from the Perspective of Noctiluca scintillans Red Tide Occurrence	Ocean Bulletin	2023-08
29	Typical Cases and Enlightenment of Risk Management of Land-based Pollution in Coastal Areas	environmental protection	2015-07

30	Progress, Problems and Policy Recommendations of Comprehensive Strengthening Marine Ecological Civilization Construction on the New Journey	environmental protection	2025-06
31	Construction of a New Marine Ecological Environment Evaluation System-Taking Zhejiang Province as an Example	Journal of Applied Oceanography	2025-08
32	Seeking Food from the Ocean: A Study on the Development of Marine Aquaculture Industry in Shantou	Guangdong Economy	2023-10
33	Research on the Evaluation and Optimization Direction of China's Deep-sea Fisheries Policy	Ocean Bulletin	2025-08
34	Estimation of Carbon Emissions from Fishing Vessels in China	Journal of Shanghai Ocean University	2010-11
35	Analysis of the Spatial and Temporal Differences of the Coordinated Development of Marine and Land Industries in China's Coastal Provinces and Cities	Statistics and Decision Making	2015-12
36	Research on the Development Model of Carbon Sink Fisheries in China	Dongyue Tribune	2011-08
37	The Present Situation, Characteristics and Development Suggestions of China's Deep-sea Aquaculture Industry	China Fishery Economy	2023-10
38	The Scientific and Economic Logic of the Development of Deep-sea Aquaculture in China	China Fishery Economy	2024-12
39	Development Ideas and Implementation Approaches of Scientific and Technological Innovation in China's Blue Granary	Journal of Fisheries Science	2019-01
40	Estimation of Carbon Sink Intensity of Algal Culture in China's Offshore Waters	Advances in Marine Science	2011-10
41	Analysis of the Situation of Marine Resources and Environmental Sustainability in China and Suggestions for Improvement	environmental protection	2015-05
42	China's Marine Carbon Sink Trading Policy, Practice and Prospect	Acta Academica Sinica	2024-03
43	Evaluation and Empirical Analysis of Marine Ecological Civilization Construction in China: A Case Study of Shandong Province	Marine and Lake Bulletin (Chinese and English)	2025-04
44	Study on the Coordinated Development Model of Marine Ecological Economic System in China	Ecological Economics	2014-02
45	The Operation and Evolution of the Collaborative Network of Marine Ranch Technology Innovation in China	Studies in the Science of Science	2022-11
46	Research on the Synergistic Development of Marine Industry Agglomeration and Carbon Emission Reduction in China	Learning and Exploration	2022-12
47	Construction and Empirical Study of Green Development Evaluation Index System of Marine Aquaculture Industry in China	marine economy	2022-01
48	Analysis of Factors Affecting Ecological Efficiency of Marine Aquaculture in China	Oceans and Lakes	2022-09
49	Research on the Evaluation of Scientific and Technological Innovation Ability of Listed Companies in China's Shipbuilding and Marine Engineering Equipment Manufacturing Industry	Journal of Jiangsu University of Science and Technology	2019-12
50	China’s Blue Granary Construction Ideas and Industrial Chain Reconstruction	Agricultural Economic Issues	2019-11
51	Analysis on the Development Status, Problems and Countermeasures of the Related Industries of China’s Blue Granary	Marine Development and Management	2019-01

52	The Development Effect, Transition Dilemma and Breakthrough Path of China's Blue Granary	Economic and Social Development	2024-12
53	Research on Monitoring and Early Warning Evaluation Technology of Marine Resources and Environmental Carrying Capacity in Tianjin	Marine and Lake Bulletin (Chinese and English)	2014-10
54	Assessment of Carbon Sink Capacity of Seawater Aquaculture in Guangdong Province under the Carbon Neutrality Target and Its Impact Effect Analysis	Strait Science	2023-03
55	The Implementation of Blue Economy Strategy Needs Dual Innovation of System and Technology	Theoretical Journal	2010-10
56	Research on the Coordinated Development of Marine and Terrestrial Industries in Shandong Province	Regional Studies and Development	2014-06
57	Changes and Exploitation of Bulk Marine Biological Resources in Shandong Province	China Marine Economy	2024-10
58	Assessment of Carbon Sink Capacity of Shellfish and Algae Cultivation in Shandong Province	China Fishery Economy	2023-02
59	Research on the Transformation and Upgrading of Qingdao Marine Economy Industry Structure: Analysis and Evaluation Based on the Efficiency of Scientific and Technological Innovation	Management Review	2018-10
60	On the Synergistic Development of Marine Culture and Marine Economy	China Agricultural Science Bulletin	2014-10
61	The Potential Carbon Source Contribution of the Benthic Food Web in the Adjacent Waters of Miaodao Archipelago in Autumn and the Consideration of Carbon Sink Fisheries	Advances in Fisheries Science	2022-10
62	China Aquaculture: Opportunities, Challenges, and Development Strategies for the Open Seas	Journal of Fisheries Science	2022-10
63	On the Construction of the Development Model of China's Blue Granary	Journal of Shandong University	2018-09
64	Research on Building a New Food Security System through Coordinated Land and Sea Development	Social Science Journals	2019-12
65	Study on the Coordinated Development Level and Influence Mechanism of Coastal Zone Composite System under the Background of Land-sea Integration: A Case Study of Wenzhou City	Advances in Geographical Sciences	2022-10
66	Assessment of Carbon Sink Capacity of Seawater Aquaculture Shellfish and Algae in Liaoning Province from 2010 to 2019	Marine Development and Management	2022-10
67	Strategic Integration Concept of Building a National Blue Granary in the Sea Area of Liaodong Greater Bay Area	Marine Development and Management	2024-06
68	Offshore Aquaculture: A New Space for Growth in Marine Food	rural economy	2019-09
69	The Composition of the Supporting Industrial System of the Blue Granary and Its Functional Positioning	Social Science Front	2015-09
70	Research on the Concept Reconstruction of Blue Granary and the Selection of Construction Mode	Dongyue Tribune	2017-06
71	Conceptual Characteristics and Evolutionary Trends of the Blue Granary	Journal of China Ocean University	2012-03
72	The Blue Granary: Construction Foundation, Challenges and Development Potential	Journal of China Ocean University	2012-03
73	Research on Carbon Emission Performance of Marine Industry in Blue Economic Zone	Regional Studies and Development	2014-06

74	Strategic Research on the Development of Marine Biological Resources in the Blue Ocean	China Engineering Science	2016-04
75	Science and Technology Innovation Supporting the Construction of Modern Marine Industry System: Mechanism, Dilemma and Countermeasures	Researching the World	2024-02
76	Assessment of Sea Area Carrying Capacity and Analysis of Obstructive Factors in Jiangsu Province	Marine and Lake Bulletin (Chinese and English)	2014-09
77	The Construction of Marine Innovation System in Canada and Its Enlightenment to China	Technological Progress and Countermeasures	2012-04
78	Research on the Construction of Environmental Pollution Control Evaluation System in Coastal Areas Based on WSR Methodology	Management Review	2021-06
79	Research on the Spatial and Temporal Differentiation of Green Production Efficiency of Marine Fisheries in China Based on SFA Model	Marine Development and Management	2023-04
80	Analysis of China's Offshore Fishery Production Efficiency Based on SFA	Agricultural Technology and Economics	2016-09
81	Research on the Dynamic Relationship between Marine Environmental Efficiency and Marine Economic Growth in China Based on SBM Model and PVAR Model	Marine and Lake Bulletin (Chinese and English)	2025-04
82	Study on Ecological Risk Assessment and Prevention Countermeasures in Guangxi Beibu Gulf Based on PSR Model	Oceanic Lakes and Marshes Bulletin	2023-12
83	Study on the Sustainable Utilization of Marine Resources in Qingdao City Based on DPSIR Model	Oceanic Lakes and Marshes Bulletin	2022-06
84	Identification and Evaluation of the Influencing Factors of Green Development Index of Island Based on DEMATEL-AHP Model	Ocean Bulletin	2024-04
85	Technical Efficiency of Seawater Aquaculture in China Based on DEA and SFA	Marine Development and Management	2018-04
86	Research on Marine Economic Vulnerability Assessment Based on CRITIC-CPM Model	Marine and Lake Bulletin (Chinese and English)	2025-01
87	Evaluation of the Habitat Status of Bohai Bay Based on the Index System Method	Journal of Water Ecology	2023-11
88	Simulation and Research on Green Development Policies of China's Marine Aquaculture Based on System Dynamics	Marine Development and Management	2023-11
89	Study on the Evaluation of the Policy Effect of Ecological Restoration in Shandong Province Coastal Zone Based on Entropy Weight TOPSIS Model	marine environmental sciences	2022-02
90	The Evaluation of the 20-year Spatio-temporal Change of Marine Environmental Quality in Xiamen Bay Based on Entropy Weight TOPSIS Model	Journal of Applied Oceanography	2024-11
91	Research on the Evaluation of the Carrying Capacity of China's Aquaculture Sea Area Based on the Entropy Weight TOPSIS Method	Marine Development and Management	2023-12
92	Research on the Spatio-temporal Evolution of Modern Marine City Based on Entropy Weight TOPSIS Method-Obstacle Degree Model-Modified Coupling Coordination Degree Model	Ocean Bulletin	2024-12

93	Study on Ecological Efficiency of Marine Fishery Based on Three-stage DEA Model: Taking Qingdao, Yantai and Weihai as Examples	fishery study	2020-12
94	Study on the Governance Efficiency of Land-based Pollution in East China Sea and Its Influencing Factors Based on Three-stage DEA-Tobit Model	Oceanic Lakes and Marshes Bulletin	2024-02
95	Carbon Sink Accounting of Seawater Shellfish and Algae Cultivation Based on the Whole Life Cycle Carbon Emission: A Case Study of Zhanjiang City, Guangdong Province	Journal of Sun Yat-sen University (Chinese and English)	2024-05
96	Evaluation of Sustainable Development of China's Marine Ecological Economy Based on Energy Value Analysis	Acta Ecologica Sinica	2017-04
97	Study on the Construction of China's Maritime Granary Based on the Experience of Japan	Dongyue Tribune	2015-04
98	Research on the Evaluation Method of Seawater Quality Based on Improved Grey Association Degree Clustering: Taking Daya Bay as an Example	Southern Fisheries Science	2024-02
99	Research on the Evaluation Index System of Offshore Ecosystem Quality Based on Multidimensional Association Analysis: Taking Bohai Bay, Yangtze River Estuary and Pearl River Estuary as Examples	China Environmental Science	2025-09
100	Evaluation of China's Marine Fishery Economic Efficiency Based on Super Efficiency SBM Model and Analysis of Influencing Factors	Ocean Bulletin	2022-06
101	Evaluation of Marine Ecological Environment Based on Bayesian Network: A Case Study of Shandong Province	Ocean Bulletin	2021-08
102	Environmental Decentralization, Marine Environmental Regulation and Marine Environmental Pollution	Statistics and Decision Making	2023-03
103	Comprehensive Evaluation of Water Quality in Coastal Waters of Major Cities around Bohai Sea	Journal of Water Ecology	2022-07
104	An Empirical Study on the Marine Economic Efficiency of Coastal Cities in the Bohai Rim Economic Circle	Statistics and Decision Making	2011-03
105	Research on the Allocation Efficiency of Green Marine Science and Technology Resources in the Bohai Rim Region	Journal of Yunnan Normal University	2020-03
106	Marine Ecological Environment Assessment and Identification of Influencing Factors in the Bohai Rim Region	Resource Science	2022-08
107	Coupling Analysis of Marine Resources and Environmental Carrying Capacity and Economic Development Level in Coastal Areas of Hebei Province	Oceanic Lakes and Marshes Bulletin	2021-12
108	Problems and Suggestions of Marine Aquaculture in Hebei Province	Hebei Fisheries	2024-04
109	Analysis of the Temporal and Spatial Evolution of Ecosystem Carbon Sources and Sinks in Hebei Province from 2000 to 2020	Journal of the Yangtze River Scientific Research Institute	2025-07
110	Study on the Countermeasures of Jiangsu Marine Economy Development from the Perspective of Dissipative Structure Theory	Journal of Nantong University	2016-11
111	Appropriateness Evaluation of Port Shorelines under the Policy Constraints of Sea Area Use Management: A Case Study of the Pearl River Estuary and Dapeng Peninsula	Science and Technology Guide	2023-02
112	The Spatial and Temporal Evolution of Carbon Emission Efficiency of Marine Fishery and Its Influencing Factors: A Case Study of the Northern Marine Economic Circle	Ecological Economics	2024-02

113	Estimation and Application of Carbon Sink in Marine Fisheries: A Case Study of Bohai Sea Region	Geographical Studies	2024-06
114	Research on Financing Efficiency of China's Marine Enterprises under the Background of Marine Power Strategy	Operations Research and Management	2025-05
115	Measurement of the Coordinated Development Level of Marine Economy and Ecological Environment	Exploration of Economic Issues	2020-12
116	Marine Industrial Policy and High-quality Development of Marine Economy: Multi-dimensional Evaluation and Nonlinear Effect Analysis	Sankei Review	2024-05
117	The Way of Seawater Culture to Help the Blue Carbon Plan	Anhui Agricultural Science	2022-01
118	Experience and Reference of the Construction of 'Blue Granary' in Foreign Countries	Journal of China Ocean University	2012-03
119	Marine Ecological Environment Governance from the Perspective of International Law: Model Transformation and China's Contribution	Journal of Jinan University	2023-12
120	Assessment of Carbon Sink Potential of Seawater Aquaculture Shellfish and Algae in Guangdong Province	Southern Fisheries Science	2012-02
121	Cultivating Sea, Raising Fish: Key Difficulties and Strategic Measures in Building a Blue Granary	developmental research	2024-06
122	Carbon sequestration capacity of marine shellfish and algae in Fujian province and its influencing mechanism	Acta Scientia Ecologicae	2024-04
123	Policy Instrument Selection and Application for Marine Ecological Environment Management in the East China Sea	Marine and Lake Bulletin (Chinese and English)	2025-09
124	Research on the Construction of Low Utility Seawater Evaluation Index System: Taking the Reclamation of Seawater in Putuo District of Zhejiang Province as an Example	Ocean Bulletin	2023-10
125	The Key Points and Countermeasures of China's Blue Granary Construction from the Perspective of the Great Food View	China Aquaculture	2024-01
126	Countermeasure Research on High-level Construction of Blue Granary in Jiangsu Province from the Perspective of Big Food	Journal of Jiangsu Ocean University	2024-05
127	The Value Implication and Practice Path of Innovation and Development of Marine Food Industry under the Concept of Big Food	Journal of Qingdao Agricultural University	2023-08
128	Development Mechanism from Carbon Sink Fishery to Blue Granary	Journal of Shanghai Ocean University	2019-11
129	Comprehensive Evaluation of the High-Quality Development Level of China's Marine Fisheries from the Perspective of Industrial Chain	Ocean Bulletin	2024-06
130	Characteristics of Carbon Emissions from Fishing Vessels in Bohai and Yellow Sea Region	Transactions of the Chinese Society of Agricultural Engineering	2022-12
131	Insurance Boosts the Construction of Blue Granary —Taking Marine Aquaculture Insurance as an Example	Fishery Information and Strategy	2015-11
132	Carbon Sink Capacity Assessment of Seawater Aquaculture Shellfish in Guangxi from 2013 to 2022	Journal of Applied Oceanography	2025-05

133	Research on the Measurement and Construction Path of Digitalization Level of Fishery under the Background of the Blue Granary Strategy	Marine Development and Management	2024-11
134	The Role of the Blue Granary in China's Food Production System and the Analysis of Construction Countermeasures	Journal of China Ocean University	2014-05
135	The Multi-dimensional Judgment of the Ecological Economic System of the Blue Granary	Chongqing Social Sciences	2016-01
136	The Strategy Selection and Its Guarantee Measures of the Expansion of the Blue Granary Space	China Fishery Economy	2013-04
137	Evaluation of the Potential of the Construction of the Blue Granary —Experience from 11 Coastal Provinces and Cities in China	Journal of China Agricultural University	2019-06
138	Sustaining the Blue Granary: Food Security under Marine Nuclear Pollution	Agricultural Economic Issues	2024-08
139	Study on the Optimization of the Related Industrial Structure of the Blue Granary	Journal of Northwest A&F University	2015-07
140	The Basic Idea and Strategy of the Construction of the Blue Granary Service Mode	World Agriculture	2017-08
141	Contribution and Forecast Study of 'Blue Granary' to China's Food Nutrition	World Agriculture	2023-03
142	Blue Granary: Research on the Dynamic Relationship between Marine Ecological Resources Carrying Capacity, Fishery Science and Technology Innovation and Fishery Economic Development—Empirical Evidence Based on Panel Data PVAR Model	Ecological Economics	2022-08
143	Blue Granary: Strategic Safeguard for National Food Security	Agricultural Economic Issues	2015-01

Table S3. All English literature references for text analysis.

No.	Title	Journal	Publication date (year-month)
1	Adjustment trend of China's marine fishery policy since 2011	Marine Policy	2020-09
2	Farmed fish welfare in Egypt: Surveying current practices and future directions for tilapia culture	Aquaculture Reports	2025-02
3	Design of a multi-use marine area off-shore the Mediterranean Sea	Ocean Engineering	2020-12
4	Major drivers of coastal aquaculture expansion in Southeast Asia	Ocean and Coastal Management	2020-09
5	Estimating the total allowable catch and management of Threadfin porgy (Evynnis cardinalis) fisheries in the northern South China Sea based on sampling surveys conducted at fishing ports	Aquaculture and Fisheries	2022-01
6	Applying a Set of Potential Methods for the Integrated Assessment of the Marine Eco-Environmental Carrying Capacity in Coastal Areas	Sustainability	2022-04
7	Effects of marine ranching policies on the ecological efficiency of marine ranching—Based on 25 marine ranching in Shandong Province	Marine Policy	2021-09

8	Measurement of carbon emissions from marine fisheries and system dynamics simulation analysis: China’s northern marine economic zone case	Marine Policy	2022-09
9	Environmental Regulation, Technological Innovation and Development of Marine Fisheries—Evidence from Ten Coastal Regions in China	Fishes	2022-01
10	Examining fishing activities based on in-situ tracking and oceanographic characteristics in Aru Sea and surroundings	Journal of Sea Research	2024-08
11	Technological Development and Fisheries Management	Reviews in Fisheries Science & Aquaculture	2014-12
12	Vulnerability of blue foods to human-induced environmental change	nature sustainability	2023-10
13	Global and Regional Determinants of Diversity in Blue Foods	Reviews in Fisheries Science & Aquaculture	2023-06
14	Blue growth and ocean governance—how to balance the use and the protection of the seas	WMU Journal of Maritime Affairs	2016-05
15	The fishery for Antarctic krill – Conflicts between industrial production, protection of biodiversity, and legal governance	Marine Policy	2025-06
16	Spatial–temporal distribution of large-size light falling-net fisheries in the South China Sea	Marine Science	2022-12
17	Seafood production in Northern Norway: Analyzing variation and co-development in aquaculture and coastal fisheries	Marine Policy	2023-07
18	Preferred Resource Spaces and Fisher Flexibility: Implications for Spatial Management of Small-Scale Fisheries	Human Ecology	2012-03
19	Analysis of the policies and constraints limiting the aquaponics industry in Portugal	Aquaculture Reports	2024-12
20	A review of global oyster aquaculture production and consumption	Marine Policy	2020-03
21	How to realize the sustainable development of mariculture industry— —Re-examine from the perspective of biased technological progress in China	Marine Policy	2021-09
22	Commercial and recreational fisher participation in marine fisheries management decision-making	Marine Policy	2025-12
23	Digital economy and economic resilience of marine fisheries in coastal cities: Based on the resource-based theory	Journal of Sea Research	2025-07
24	The rise of aquaculture by-products: Increasing food production, value, and sustainability through strategic utilization	Marine Policy	2018-01
25	Environmental problems and regulation in the aquaculture industry. Insights from Norway	Marine Policy	2018-08
26	Management of China's capture fisheries：Review and prospect	Aquaculture and Fisheries	2019-05
27	A collective approach to Pacific islands fisheries management: Moving beyond regional agreements	Marine Policy	2010-12
28	Managing marine aquaculture by assessing its contribution to ecosystem services provision: The case of Mediterranean mussel, Mytilus galloprovincialis	Ocean and Coastal Management	2024-10
29	Seventy years of small-scale fisheries in Uruguay: Trends, challenges, and future directions	Ocean and Coastal Management	2025-07
30	The marine economy of the United Kingdom	Marine Policy	2020-03
31	Nuts and bolts in fisheries management—a technological approach to sustainable fisheries?	Marine Policy	2004-04
32	Optimal effort, fish farming, and marine reserve in fisheries management	Aquaculture and Fisheries	2023-05

https://doi.org/10.3390/xxxxx

33	Persistence of fisheries production: a disaggregated analysis in 31 OECD Countries	Marine Policy	2024-05
34	Fishery Resources, Ecological Environment Carrying Capacity Evaluation and Coupling Coordination Analysis: The Case of the Dachen Islands, East China Sea	Marine Science	2022-05
35	Need for integrated analysis and management instruments to attain sustainable fisheries in Vietnam	Sustainability of Water Quality and Ecology	2014-12
36	Balancing local ownership with foreign investment in a small island fishery	Ocean & Coastal Management	2007-03
37	Status of aquatic organisms' resources and their environments in Yangtze River system (2017–2021)	Aquaculture and Fisheries	2023-07
38	Effectiveness of formal institutions in managing marine fisheries for fisheries development: A case study of a coastal commune sustainable in Vietnam	Ocean & Coastal Management	2016-12
39	Food for thought: A realistic perspective on the potential for offshore aquaculture in the Dutch North Sea	Journal of Sea Research	2022-12
40	The evolution of food and nutrition supply patterns of marine capture and mariculture in China and its transformation coping strategies	Marine Science	2024-10

Supplementary Materials Section S2

Pearson Correlation Coefficient Test Results

To further investigate the degree of linear correlation among the indicators, this study calculated the Pearson correlation coefficients for all pairwise combinations of the 28 tertiary indicators. The data sources remain consistent with those previously described (panel data of 11 coastal provinces and cities from 2010 to 2022), with all indicators standardized using the Min-max normalization method. Table 2 presents the correlation coefficients for a selection of representative indicator pairs, encompassing both intra-dimensional and cross-dimensional combinations.

Table S4. Pearson Correlation Coefficients for Selected Tertiary Indicator Pairs.

Indicator A	Indicator B	Correlation Coefficient
Number of Motorized Fishing Vessels (vessels)	Motorized Fishing Vessel Power (kW)	0.75
Number of Motorized Fishing Vessels (vessels)	Fishing Gear Value (10,000 yuan)	0.65
Marine Aquaculture Area (ha)	Marine Shellfish Production (tons)	0.70
Marine Aquaculture Area (ha)	Marine aquaculture output value (10,000 yuan)	0.68
Marine Fish Production (tons)	Marine Fishing Output Value (10,000 yuan)	0.72
Marine Shrimp and Crab Production (tons)	Other Marine Product Production (tons)	0.45
Fisheries-related population (persons)	Professional Personnel (persons)	0.78
Professional Personnel (persons)	Aquatic Technology Extension Personnel (persons)	0.55
Coastline Index	Island Index	0.60
Marine Algae Production (tons)	Marine aquaculture output value (10,000 yuan)	0.62
Number of Fisheries Law Enforcement Agencies	Number of Fisheries Management Personnel	0.73
Number of Fisheries Management Personnel	Economic losses from fishery disasters (10,000 yuan)	-0.30
Land-Based Pollutants Discharged into Sea (tons)	Red Tide Disasters (hectares)	0.50

Per capita net income of fishermen (10,000 yuan)	Marine aquaculture output value (10,000 yuan)	0.55
Shallow Coastal Tidal Flat and Bay Cultivable Area (hectares)	Marine Shellfish Production (tons)	0.58

As shown in Table A3, the vast majority of correlation coefficients between indicator pairs fall within the range of 0.3 to 0.7, indicating moderate or weak correlation. Certain indicators within the same dimension exhibit relatively higher correlations due to reflecting similar economic or productive activities. For instance, the correlation coefficient between "Number of Motorized Fishing Vessels" and "Motorized Fishing Vessel Power" is 0.75, and between "Fisheries-related population" and "Professional Personnel" it is 0.78. However, all coefficients are below the empirical warning threshold of 0.8. Negative indicators (e.g., economic losses from fishery disasters) generally show weak negative or near-zero correlations with other indicators, which aligns with expectations.

A statistical summary of all pairwise correlation coefficients for the 28 indicators (a total of 378 pairs) reveals the following distribution:

(1) Number of pairs with an absolute value greater than 0.8: 0

(2) Number of pairs with an absolute value between 0.7 and 0.8: 6, primarily concentrated within the "Production Resources" and "Human Resources" dimensions

(3) Number of pairs with an absolute value between 0.5 and 0.7: 42; The remaining 330 pairs have absolute correlation coefficients below 0.5

This distribution pattern indicates that while some correlation exists among indicators within the same dimension, the overall degree of multicollinearity is low and does not reach a level that would compromise the results of the comprehensive evaluation. For the few indicator pairs with correlations approaching 0.8 (e.g., "Number of Fisheries Law Enforcement Agencies" and "Number of Fisheries Management Personnel"), the research team conducted a redundancy review based on the theoretical framework. Although numerically correlated, these indicators conceptually represent different facets—"institutional setup" versus "personnel allocation"—both falling under the governance dimension. Retaining both contributes to a more holistic characterization of administrative management capacity. Should future research prioritize parsimony, consolidation or removal could be considered. However, given this study's aim to maintain the integrity of the evaluation system, both indicators were retained.

In summary, the Pearson correlation coefficient analysis further validates the statistical robustness of the constructed indicator system, confirming the absence of severe multicollinearity issues that could significantly impact the weight calculation and obstacle diagnosis.
